# Surface‐modified nanosilica–chitinase (SiNp‐Chs)‐doped nano enzyme conjugate and its synergistic pesticidal activity with plant extracts against armyworm *Spodoptera litura* (Fab.) (Lepidoptera: Noctuidae)

**DOI:** 10.1049/nbt2.12004

**Published:** 2021-02-19

**Authors:** G. Narendrakumar, S. Karthick Raja Namasivayam

**Affiliations:** ^1^ Department of Biotechnology School of Bio and Chemical Engineering Sathyabama Institute of Science and Technology Chennai Tamil Nadu India; ^2^ Centre for Bioresource & Development (C‐BIRD) School of Bio and Chemical Engineering Sathyabama Institute of Science and Technology Chennai Tamil Nadu India

## Abstract

A laboratory experiment was conducted to evaluate enhanced pesticidal activity of silica nanoparticles‐doped chitinase nano enzyme conjugate against an economically important insect pest *Spodoptera litura* (Fab.) (Lepidoptera; Noctuidae). Silica nanoparticles were synthesized by hydrolysis and condensation of precursor tetraethylorthosilicate (TEOS) followed by functionalization with functioning agent 3‐aminopropyltriethoxysilane. Functionalized silica nanoparticles thus acquired were doped with chitinase enzyme produced by *Serratia marcescens* SU05. Doped nanosilica–chitinase nano enzyme conjugate was loaded with pesticidal plant extracts to study the improved pesticidal activity. Synthesized nano enzyme conjugate revealed high stable, monodisperse spherical nanoparticles and exhibited effective loading with respective plant extracts. Nano enzyme conjugates and plant extracts loaded with nano enzyme conjugate recorded high rate of mortality against the larval instars and brought about a distinct effect on the life stage parameters of *S.litura*. Non‐target toxic effect of nano enzyme conjugate was carried out by determination of lethality and changes in protein profiling against brine shrimp (*Artemia salina*) that shows less lethality and no distinct changes in protein profiling which suggest the effective utilization of silica nanoparticles doped chitinase as an insecticidal agent against economically important insect pests associated with various crops.

## 1 INTRODUCTION

Agriculture is the largest monetary sector that plays an imperative role in the socioeconomic growth of the country, and there has been a gradual slump in its contribution to the GDP of the country. The total tillable territory in India is 15,73,50,000 km^2^, that delineate over 52.92% of the inclusive land of the country. Cultivatable land in India was curtailed due to continuous strain from an ever‐increasing number of dwellers and growing urbanization. Among the various constraints in agriculture, insect pests play a vital role and the control of insect pests heavily depends on chemical pesticides [1]. Environmental pollution instigated by pesticides and their squalor products is a major biological problem [2]. The major ecological apprehension in the use of pesticides is their capacity to trickle from soil and the ground water blemish [3]. They would endure on the top soil where it could accrue to noxious levels in the soil and become detrimental to microorganisms, plants, wildlife and man [4]. Spraying of pesticides in soils has become lethal to bacteria, fungi, protozoa, earthworms and arthropods that are vivacious to bionetworks, because they prime both the organization and utility of ecological system [5].

*Spodoptera litura* (Fabricius) (Lepidoptera: Noctuidae), a polyphagous insect of pluralistic dissemination, has a substantial host array of more than 150 host species [6] and is contemplated vital in numerous stretches, countries including Indian subcontinent and Asian countries [7]. It is commonly known as Tropical armyworm; Cluster caterpillar; Cotton leafworm; Tobacco cutworm. *Spodoptera litura* (*S. litura*) is a defoliating insect pest that distresses the harvest of various cultivated cash crops, vegetables, fruits, weeds and ornamental plants by nourishing sociably on leaves and it instigates outsized monetary fatalities in crop plants [8]. The controlling of *S.litura* to safeguard the steady and extraordinary productivity of crops is a prodigious encounter in agrarian turf and therefore, pesticide treatment is broadly adept for its control [9]. There is extensive apprehension over adverse influence of pesticides on environmental condition owing to accretion of pesticide drugs in addition to rise of pesticide resistance in the insects [10]. Occasionally, when chemical pesticides were applied on diverse groups of predators that invaded the environment, it resulted in pest resurrection and outburst of subordinate pests [11].

Nanotechnology in agriculture played a pioneering role in creating sustainable agriculture. It works at the atomic, molecular and sub‐molecular levels [12]. Nanomaterials possess important properties of self‐assembly, stability, specificity, encapsulation and biocompatibility. Novel approach to the control of insect pests is the use of DNA‐tagged gold nanoparticles that effective against *S.litura* reported by Chakravarthy et al. [13]. Application of nanotechnology in agriculture includes plant breeding, precision agriculture, disease control, biotechnology genetics, fertilizer technology and allied fields. If farmers are given good understanding of agricultural production system, the application of nanotechnology has bright prospects like nanoformulations of fertilizer, surveillance and control of pests and diseases, mechanism of host parasite interaction at molecular level, development of new generation pesticides and their careers, preservations and packaging of food additives, strengthening of natural fibres, removal of contaminants from water and soil, increasing the shelf life of flowers and vegetables, clay‐based nano‐sensors for precision water management, reclamation of salt affected soils, stabilization of erosion‐prone surfaces [14]. Precision farming, antimicrobial nano‐materials for plant pathogens and development of nano‐pesticides are modern approaches in agriculture. Nano‐farming, nano‐food and nano‐packaging are the features of nanotechnology in food industry. Nano‐sensors are applied in smart gene delivery system and in detection of pathogen. Insect pests, weed and fungi are to be managed by the use of nano‐biopesticides, herbicides and fungicides [15]. These develop draught and pest resistant crops. Application of nanotechnology improved food quality and food safety improves processing and nutrition [16]. Nanotechnology offer eco‐friendly alternatives for plant disease management [17,18].

Certain carbon nano tubes (1 nm) have the tremendous potential to protect host plants from insect pests [19]. Enhanced insecticidal activity of nanoparticles loaded pesticides, insecticides and insect repellents have reported [20, 21, 22, 23]. Nanotechnology principles can be used to deliver DNA and other desired chemicals into plant tissues for protection of host plants against insect pests. Porous hollow silica nanoparticles (PHSNs) loaded with validamycin (pesticide) can be used as efficient delivery system of water‐soluble pesticide for its controlled release. Such controlled release behaviour of PHSNs makes it a promising carrier in agriculture, especially for pesticide‐controlled delivery whose immediate as well as prolonged release is needed for plants [24]. Oil in water (nano‐emulsions) was useful for the formulations of pesticides and these could be effective against the various insect pests in agriculture. Similarly, essential oil‐loaded solid lipid nanoparticles were also useful for the formulations of nano‐pesticides [16,17,25]. Nanosilica, a silica product, can be effectively used as a nanopesticide. Barik et al. (2008) [1] reviewed the use of nano‐silica as nano‐insecticide. The mechanism of control of insect pest using nano‐silica is because insect pests used a variety of cuticular lipids for protecting their water barrier and thereby prevent death from desiccation. But here, the nanosilica particles when applied on plant surface, cause death by physical means of insects by being absorbed into the cuticular lipids. Modified surface charged hydrophobic nano‐silica (∼3‐5 nm) could be successfully implemented to manage a variety of ectoparasites of animals and agricultural insect pests. The insecticidal activity of polyethylene glycol‐coated nanoparticles loaded with garlic essential oil against adult *Tribolium castaneum* insect found in stored products [26].

Pesticidal activity of silver nanoparticles [20], zinc oxide and titanium dioxide [7]; chitosan nanoparticle‐loaded fungal metabolites [27,28], nanostructured alumina [29,30], Zinc oxide ZnO [24], nano‐encapsulated essential oils from *Zanthoxylum rhoifolium* [3] revealed the nanotechnology based effective control of economic important insect pests associated with a wide range of crops. However, very few works available on the nano enzyme conjugate mediated pesticidal activity against major lepidopteron insect pests. With this object, we proposed to study the chitinase enzyme‐doped silica nanoparticle‐based pesticidal activity against *S.litura* (Fabricius) (Lepidoptera: Noctuidae). *S.litura* is an important defoliator associated with many economically important crops. Management of this pest is highly complicated because of its insecticide resistance. Chitinases (EC 3.2.1.14) can catalyse the hydrolysis of chitin to its monomer *N*‐acetyl‐d‐glucosamine produced by a wide range of microorganism Chitinases can be exploited for their use in control of fungal other insect pathogens of plants and insects pests by degrading chitin—a structural component of cells [22]. Silicon dioxide nanoparticles, also known as silica nanoparticles or nanosilica, are the basis for a great deal of agriculture research due to their stability, high efficacy against insect pests, best biocompatibility and ability to be functionalized with a range of molecules and polymers [29]. With this object, this study aimed to develop silica nanoparticle‐loaded chitinase nanoformulation and its enhanced pesticidal activity against *S.litura* that would suggest possible utilization of nano formulated enzyme as a green pesticidal agent. The novelty of this work was preparing nano monodisperse silica nanoparticles coupled with microbe‐derived chitinase and its effect was tested in the target and non‐targeted organisms.

## 2 MATERIALS AND METHODS

### 2.1 Bacterial strain and growth condition

The bacterial strain were isolated from the soil of prawn culture farms near Chennai (12°48′30.8″N 80°14′50.6″E), India, the organisms were purified, screened and characterized for the maximum estimation of the chitinase enzyme [22].

### 2.2 Production of chitinase by solid‐state fermentation

#### 2.2.1 Inoculum preparation

Bacterial culture was inoculated from nutrient agar slant into 100 ml of primary inoculum media—minimal media with 0.5 % colloidal chitin, incubated under shaking condition at 35°C for 48 h.

#### 2.2.2 Preparation of substrate

Fourth instar larvae of *S.litura* was used as the substrate in this study. Larvae were collected from laboratory stock culture. About 100 g of healthy, live fourth instar of *S.litura* was transferred to the beaker containing hot water. After the heat treatment, the contents were filtered through muslin cloth and, the collected larvae kept on the filter paper to remove the moisture content.

### 2.3 Optimization of incubation time for enzyme production

The 0.5 moisture of heat‐treated larvae was added along with 2.5 mg of bacterial suspension and incubated at different time intervals from 12 to 96 h. The enzyme activity was tested and maximum productivity was estimated.

### 2.4 Optimization of process condition for the chitinase production by response surface methodology (RSM)

#### 2.4.1 Experimental design and optimization studies

Central composition design (CCD) was used for optimizing the parameters for the production of chitinase using *S.litura* larvae as the substrate by solid‐state fermentation (SSF). Known quantity (100 g) of *S.litura* heat‐treated larvae were transferred to the 250 ml of conical flask, sterilized by autoclaving. After sterilisation, the moisture content of the medium was adjusted by adding sterile distilled water. Bacterial inoculum was added to the respective flasks and maintained under static condition at different incubation time. The Design of Experiment (DoE) given by Design Expert—version 7.0.0 (Table 1) was used to perform the runs.

**TABLE 1:** Design summary of RSM for solid‐state fermentation

Factor	Name	Low			High	
		Coded		Actual	Coded	Actual
X_1_	Moisture content	‐1		0.1	1	1
X_2_	Inoculum size	‐1		0.1	1	5
X_3_	Incubation time	‐1		12	1	96
Name	Unit	Obs	Min	Max	Mean	Std. Dev.
Enzyme activity	IU/ml	20	11.7	41.3	23.828	11.431
Pesticidal activity	%	20	18.4	85.4	47.061	22.787

### 2.5 Extraction of chitinase

After the incubation period, the conical flasks with the substrate was filled with 25 ml of sterile distilled water, kept under shaking condition for 1 h at 30°C.Contents was filtered through Whatman's No.1 filter paper followed by centrifugation at 10,000×*g*. Collected supernatant was used as the source of enzyme.

### 2.6 Chitinase assay

The chitinase activity was assayed using DNS method (Miller, 1959), absorbance of the supernatant was measured at 540 nm. One unit of chitinase activity was defined as the amount of enzyme that liberates 1 mol of *N*‐acetylglucosamine per minute under described conditions.

### 2.7 Silica nanoparticles synthesis

Silica nanoparticles were synthesized by hydrolysis and condensation of precursor tetraethylorthosilicate (TEOS) with water–ethanol mixture, ammonium hydroxide. The mixture was kept under stirring under magnetic stirrer for three hours.

### 2.8 Optimization of parameters

By optimizing the concentration of respective reagents, the size of the nanoparticles varied accordingly resulting in effective nanoparticles. This process was performed by central composite design using response surface methodology (Table 2). Ethanol–water mixture (10, 20, 30), ammonium hydroxide (1, 2, 3) and tetraethylorthosilicate (1, 3, 5) in different combinations as predicted by DoE, the analysis was performed using scanning electron microscopy (SEM) to identify the size of nanoparticle.

**TABLE 2:** Design summary of RSM for synthesis of silica nanoparticle

Factor	Name		Low		High	
			Coded	Actual	Coded	Actual
A	Ethanol–water mixture		‐1	10	1	30
B	Ammonium hydroxide		‐1	1	1	3
C	Tetraethylorthosilicate		‐1	1	1	5
Name	Units	Obs	Min	Max	Mean	Std. Dev.
Size	Nm	20	70	340	199.9	102.881

### 2.9 Functionalization of silica nanoparticles doped chitinase

Sol‐gel method was used for the synthesis of functionalized silica nanoparticles doped chitinase. About 50 mg of prepared nanosilica was suspended in 50 ml of distilled water contain ethanol (1:2 ratio) followed by the addition of 2.5 ml of 1% ammonium hydroxide, 0.05 g of lyophilized enzyme and 3 ml of 1% functionalization agent 3‐amino propyl triethoxy silane (APS).The mixture was kept under stirring under magnetic stirrer for three hours. To remove non‐functionalized silica nanoparticles, the mixture was centrifuged at 3000 rpm for 30 min followed by dissolving in ethanol.

### 2.10 Characterization

#### 2.10.1 Fourier transform infrared spectroscopy (FT‐IR)

Primary characterisation of nanosilica‐doped chitinase was carried out by Fourier transform infrared spectroscopy (FT‐IR) that is used to analyse the changes in functional groups. Samples for analysis were prepared by uniform mixing of dried samples with potassium bromide at suitable ratio, compressed under specific pressure load to form discs. Discs were scanned using Bruker Optic GmbH Tensor 27 in the range of 400–4000 cm^−1^.

#### 2.10.2 Field emission scanning electron microscopy (FESEM) and energy dispersive atomic spectroscopy (EDAS)

By adopting field emission scanning electron microscopy (FESEM) methods, the study of characterisation of particle shape and size morphology is evolved. The above modus operandi is carried out with the support of focused electron with a high vacuum contained in SUPRA 55‐CARL ZEISS (Germany) that has a magnification range of 35–10,000, resolution 200 A°, acceleration voltage 19 kV.

### 2.11 Insect collection and maintenance

Pesticidal activity was studied against third and fourth instars of *S.litura*. Larvae were collected from the laboratory stock culture and reared on the castor leaves in the stainless steel trays covered with muslin cloth for aeration less than 28°C, 80% relative humidity and 12 L.12 D photoperiod.

### 2.12 Bioassays

Food and contact toxicity assays were used to study pesticidal activity of free silica nanoparticles and silica‐doped chitinase nanoformulation.

#### 2.12.1 Food toxicity assay

Fresh castor leaves were sprayed with different concentrations of free and respective nano‐formulation using ultra low volume sprayer, allowed to dry under laminar air flow chamber. Treated dried leaves were transferred to the plastic container with meshed lid and moist filter paper at the bottom to provide aeration and humidity, respectively. Twenty five larvae of third and fourth instars were introduced into the container after 6‐h starvation. Daily observation was made to record cumulative mortality and developmental influence. Containers were incubated at ambient temperature (29^o^C). Triplicates and control were maintained for each treatment.

#### 2.12.2 Contact toxicity assay

Pesticidal effect of free silica nanoparticles (F‐SiNps) and silica nanoparticle‐doped chitinase was also studied by contact toxicity. Respective nanoformulation was dispersed in suitable dispersion medium and the aliquots thus obtained were used for the contact toxicity assay. Twenty five larvae of respective instar was separately dipped in the respective dosages of nanoformulation for 1 min, transferred to the plastic container. Fresh castor leaves were supplied regularly. Containers were incubated at ambient temperature (29^o^C). Daily observation was recorded to determine cumulative mortality, developmental influence, lethal time 50 (LT_50_) and lethal concentration 50 (LC_50_)

### 2.13 Synergistic activity of SiNp‐Chs with biopesticidal plant extracts

Synergistic activity of biopesticidal plants like *Azadirachta indica, Adhatoda vasica, Leucas aspera* and *Curcuma longa* with SiNp‐Chs was also studied to determine enhanced insecticidal activity against *S.litura*.

#### 2.13.1 Collection of plant materials

Rhizome of *Curcuma longa*, leaves of *Azadirachta indica, Adhatoda vasica* and *Leucas aspera* were collected from home garden in a sterile polythene bag and brought to the laboratory for further studies.

#### 2.13.2 Preparation of extracts

Respective collected plant material was washed with sterile distilled water followed by shade drying at room temperature. After drying, finely ground into powder using domestic mixture and stored in an airtight plastic sampling bags for further studies. Extraction was carried out by the modified method of Hussaini and Mahasneh [10]. Extraction of the respective plant material (10 g) was done twice with 100 ml of 99 % ethanol at room temperature for 48–72 h. After the incubation period, the contents were filtered through Whatmann No.1 filter paper, the collected filtrate was concentrated in rotatory vacuum drier at 40°C. Concentrated extract thus obtained was collected in screw cap vial and used for further studies.

#### 2.13.3 Preparation of plant extract‐loaded SiNp‐Chs

About 1.0 ml of LC_50_ concentration of respective (reconstituted from original stock) plant extract was suspended in 100 ml distilled water–ethanol mixture containing 0.1 g of functionalised SiNp‐Chs, the mixture was stirred under magnetic stirrer at room temperature for 2 h to obtain the homogenized mixture followed by centrifugation at 10,000×*g*. Collected pellet was washed with ethanol–water mixture and lyophilized.

#### 2.13.4 Evaluation of enhanced pesticidal activity

Enhanced pesticidal activity of SiNp‐Chs loaded with respective plant extract was carried out against 3rd and 4th instars larvae. Respective plant extract‐loaded SiNps‐Chs with different concentrations were dissolved in ethanol–water mixture to produced aliquots as 10, 25, 50, 75 and 100 and used for food and contact toxicity assays as described earlier. Evaluation of pesticidal activity was determined by total development days, cumulative mortality, lethal time 50 (LT_50_) and lethal concentration 50 (LC_50_).

### 2.14 Evaluation controlled or sustained release study

Known concentration of SiNp‐Chs loaded with respective plant extracts was dissolved in 5 ml of ethanol‐water mixture in a centrifuge tube, kept in orbital shaker at room temperature. Centrifugation was done every 1h at 10,000×*g* for 10 min. Supernatant was collected in separate sterile centrifuge tube, filtered through syringe filter (0.4 µm), collected in sterile screw cap vial and used for larvicidal activity against larval instars of *S.litura* adopting both food and contact toxicity tests as described above. Three replicates and control were maintained. Triplicates and control were maintained for each treatment. Determination of mortality with respect to time period against tested instar of revealed the release of metabolites.

### 2.15 Brine shrimp (*Artemia salina*) lethal toxicity testing

Non‐target effect of free silica nanoparticles, free chitinase and silica nanoparticle‐loaded chitinase against brine shrimp (*A.salina*) was studied by determination of mortality, lethal time 50 (LD 50) and changes in protein profile.

#### 2.15.1 Brine shrimp (*Artemia salina*) collection and maintenance

Brine shrimp *A.salina* cysts were purchased and maintained in the laboratory conditions by the modified method of McLaughlin et al. [12]. Toxicity assay was done in 1 L glass jar. Known amount of *A.salina* collected cysts were transferred to the rearing glass jar with 3.5% of artificial sea water followed by constant aeration for 48 h at 29°C. After hatching, the free floating nymphs were collected and used for the lethal toxicity testing.

#### 2.15.2 Toxicity testing

A 96‐well plate assay was used to confirm toxicity or lethality of the respective formulation [23]. Hatched nymphs [20] were transferred to the each well of the 96‐well plate followed by the addition of 0.1 ml of different aliquots (prepared from original stock) of respective formulation. Triplicates were maintained in each treatment. Seeded plate was incubated at ambient temperature (28^o^C) for 24 h. After the incubation period, the live nymphs were counted in the respective treatment using stereomicroscope (Leica M205 A). Lethality in percentage was calculated by the following formula

Lethality(%)=Test−control⁄Test×100

The toxicity of respective nanoformulation was determined from the 50% lethality dose (LD_50_) using Probit analysis (Finney 1949).

### 2.16 Effect of nanoformulation on protein profile of *A. salina*

Nanoformulation‐mediated toxic effect on protein profile of *A.salina* was evaluated by protein profiling study. Surviving nymphs from the respective treatment groups were collected after the incubation period followed by homogenization using pestle and mortar. Homogenate thus obtained from the respective treatment group were transferred to the centrifuge tubes (5 ml), centrifuged at 15,000×g for 15 min at 4°C. The collected supernatant was used for protein profiling studies by SDS‐PAGE analysis.

#### 2.16.1 SDS‐PAGE

The gel was polymerized from a mixture of 30% acrylamide 0.8% methylene bisacrylamide, 1.5 M tris hydrochloride (pH 8.8), 25 ml of distilled water, 0.5% of *N*,*N*,*N*’,*N*’ tetramethylene diamine, 1.5 ml of ammonium persulphate. The crude protein extract of the control and treated samples were solubilized in sodium phosphate buffer (pH 7.5), centrifuged at 10,000×*g* for 2 min. About 50 µg of the crude protein was loaded onto the gel and run at 50 V till the sample cross the stacking layer. Electrophoresis was carried out at 10°C with 0.05 M tris‐glycine buffer (pH 8.3). The protein bands were visualized after staining with Coomassie brilliant blue followed by destaining with methanol and acetic acid.

## 3 RESULTS AND DISCUSSION

### 3.1 Identification and characterization of bacterial strain

*Serratia* sp. was isolated from the prawn culture farms near Chennai, India, the organisms were purified, screened and characterized for the maximum estimation of the chitinase [22].

**FIGURE 1:**
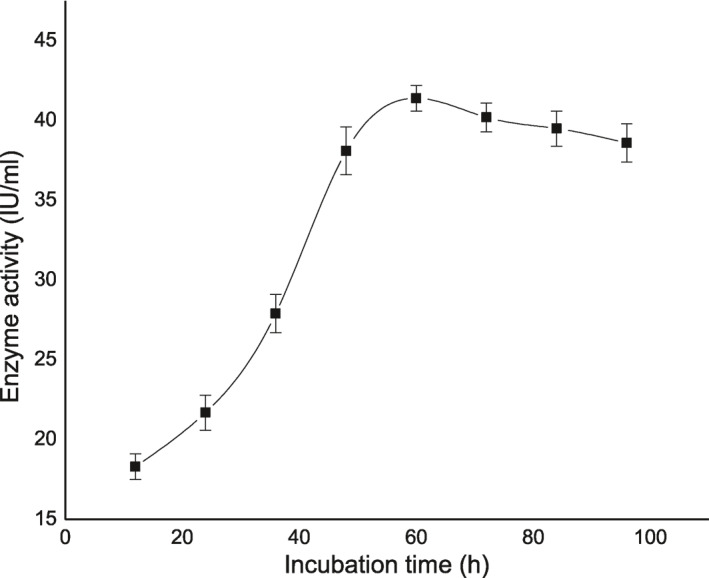
The effect of incubation time on enzyme activity

### 3.2 Effect of incubation time on enzyme activity

The pure culture of isolated organism *Serratia* was subjected to identify the better incubation time by keeping the other two parameters as 0.2 as moisture content and 2.5 mg as inoculum size. The incubation time was determined at 60 h and after 60th hour the enzyme activity seems to be stable (Figure 1).

### 3.3 Optimization of process parameters on chitinase production by SSF using RSM

The media for solid‐state fermentation was optimized various cultural parameters such as inoculum size, incubation time and moisture content using response surface methodology (RSM) using Central composite design (CCD). The model used was quadratic, and 20 runs trial was performed. The enzymatic activity and pesticidal activity were calculated for the DoE that was given by Design expert software. The response was estimated by chitinase and pesticidal activity was displayed as contour plot and surface plot (Table 3).

(1) $$Enzyme\;activity=40.448+3.275X_{1}-0.316X_{2}+0.219X_{3}-0.126X_{1}X_{2}+0.052X_{1}X_{3}+0.32X_{2}X_{3}-7.612X_{1}^{2}+8.215X_{2}^{2}-8.711X_{3}^{2}$$

**TABLE 3:** Design of Experiments with response (enzyme activity and pesticidal activity)

Run	X_1_	X_2_	X_3_	Enzyme activity		Pesticidal activity	
				Actual	Predicted	Actual	Predicted
				IU/ml		%	
1	0.1	0.1	96	19.4	19.4	38.8	39.5
2	0.1	5	96	18.3	18.3	36.6	36.6
3	1	5	12	12.6	12.68	25.2	25.25
4	‐0.2	2.5	54	24.5	24.42	49	49.10
5	0.5	2.5	54	40.1	40.72	82.6	80.31
6	0.5	2.5	54	40.7	40.72	85.4	80.31
	1	5	96	11.7	11.69	18.4	19.91
8	0.1	0.1	12	19.2	19.31	38.4	37.52
9	0.5	6.7	54	17.0	16.97	34.0	33.02
10	1	0.1	12	12.9	12.91	25.8	26.56
11	0.5	2.5	‐16.	16.5	16.44	33.0	33.05
12	1	0.1	96	13.1	13.23	26.2	26.36
13	0.5	2.5	124.6	15.8	15.71	31.6	30.52
14	0.5	2.5	54	41.3	40.72	70.5	80.31
15	0.5	2.5	54	40.2	40.72	79.2	80.31
16	0.5	2.5	54	41.1	40.72	82.2	80.31
17	0.5	2.5	54	40.9	40.72	81.8	80.31
18	1.3	2.5	54	13.5	13.41	27	25.84
19	0.5	‐1.5	54	18.1	17.98	36.2	36.15
20	0.1	5	12	19.6	19.59	39.2	39.84

**FIGURE 2:**
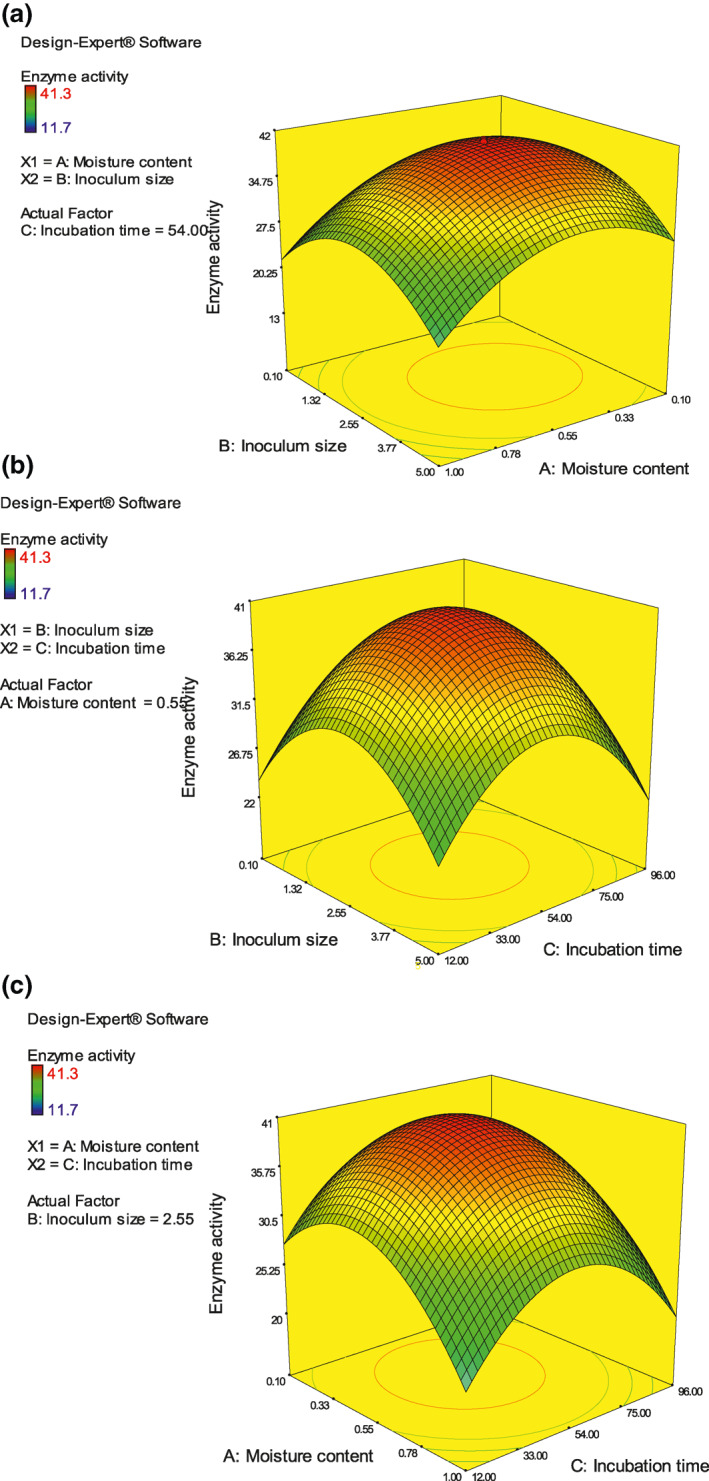
(a) B: Inoculum size versus A: Moisture content. (b) B: Inoculum size versus C: Incubation time. (c) A: Moisture content versus C: Incubation time, the response was evaluated as enzyme activity

The effect of moisture content (a) with respect to Inoculum size (b) express that as the moisture increases the enzyme activity decreases significantly (Figure 2a), the incubation time plays an significant role in the enzyme activity with respect to the inoculum size (Figure 2b), at an optimum incubation time of ‐50 to 55 h maximum activity was observed. As the moisture content increases the as the incubation time the enzyme activity was decreasing (Figure 2c). This confirms that the key variable is moisture content (a) that influence both incubation time and inoculum size The *R* ^2^ value was 98.99% compared with adjusted *R* ^2^ of 98.90% with respect to the predicted *R* ^2^ of 97.90% confirms the fitness of the test Table 4)

(2) $$Pesticidal\;activity=79.71-6.93\;X_{1}-1.037\;X_{2}-0.84\;X_{3}-0.90\;X_{1}X_{2}-0.5456\;X_{1}X_{3}-1.28\;X_{2}X_{3}-14.94\;X_{1}^{2}-16.162X_{2}^{2}-17.156X_{3}^{2}$$

**TABLE 4:** ANOVA for SSF (enzymatic and pesticidal activity)

	Enzymatic activity				Pesticidal activity			
Source	Sum of squares	Mean square	F value	*p*‐Value Prob > F	Sum of squares	Mean square	F value	*p*‐Value Prob > F
Model	2612.11	290.23	2315.7	<0.0001	10,242.9	1138.10	79.8425	<0.0001
X_1_	146.6428	146.6428	1170.039	<0.0001	657.138	657.138	46.1009	<0.0001
X_2_	1.362617	1.362617	10.87209	0.0081	14.4709	14.4709	1.01519	0.03374
X_3_	0.654787	0.654787	5.224434	0.0453	9.60264	9.60264	0.67366	0.04309
X_1_ X_2_	0.127735	0.127735	1.019178	0.03365	6.62738	6.62738	0.46493	0.05108
X_1_ X_3_	0.02187	0.02187	0.174499	0.06850	2.39313	2.39313	0.16788	0.06906
X_2_ X_3_	0.85853	0.858532	6.850083	0.0257	13.2057	13.2057	0.92643	0.03585
X_1_^2^	822.4132	822.4132	6561.898	<0.0001	3167.97	3167.97	222.245	<0.0001
X_2_^2^	971.6813	971.6813	7752.883	<0.0001	3760.12	3760.12	263.787	<0.0001
X_3_^2^	1093.076	1093.076	8721.467	<0.0001	4239.12	4239.12	297.391	<0.0001
Residual	1.253316	0.125332			142.543	14.2543		
Lack of Fit	0.084983	0.016997	0.072738	0.9940	8.13514	1.62703	0.06052	0.9960
Pure Error	1.168333	0.233667			134.408	26.88167		
Cor total	2613.364				10,385.4			
Std. Dev.	0.354022	*R* ^2^		0.989952	Std. Dev.	3.775493	*R* ^2^	0.986275
Mean	23.828	Adj *R* ^2^		0.989089	Mean	47.061	Adj *R* ^2^	0.973922
C.V. %	1.48574	Pred *R* ^2^		0.979099	C.V. %	8.022552	Pred *R* ^2^	0.974872
PRESS	2.353515	Adeq precision		115.9445	PRESS	260.9621	Adeq precision	22.62374

**FIGURE 3:**
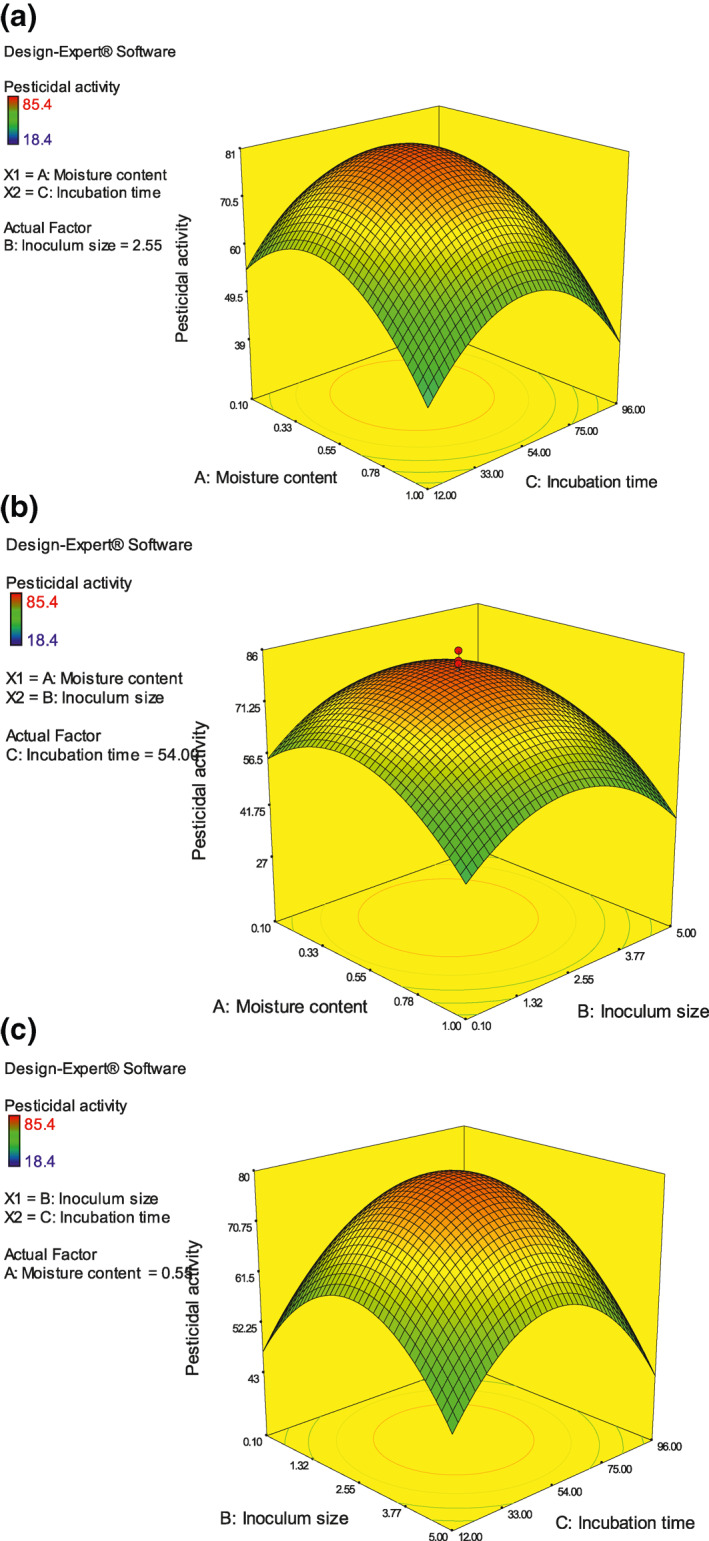
(a) A: Moisture content versus B: Inoculum size. (b) B: Inoculum size versus C: Incubation time. (c) A: Moisture content versus C: Incubation time, the response was evaluated as pesticidal activity

Effect of parameters on the pesticidal activity against third instar of *S.litura* was studied by RSM (Figure 3a–c) (Table 4) that revealed maximum pesticidal activity was recorded in chitinase enzyme produced under optimum condition, as described above. These results were also correlated with the enzyme activity, this confirms that the relativeness between the enzyme activity and pesticidal activity. The *R* ^2^ value was 98.62% compared with adjusted *R* ^2^ of 97.39% with respect to the predicted *R* ^2^ of 97.48% confirms the fitness of the test.

### 3.4 Free silica nanoparticles synthesis

#### 3.4.1 Response surface methodology

Synthesis of silica nanoparticles was done by changing the concentration of water ethanol, NH_4_OH and TEOS that was optimized under standard condition by RSM (Table 5). Optimum condition for the synthesis was determined by changing the reaction mixture into white suspension uniform particles with nano range as mentioned below.

**TABLE 5:** Design of Experiments with response (nanoparticle synthesis)

Run	A:Ethanol‐water Mixture	B:Ammonium Hydroxide	C:Tetraethylortho Silicate	Size	
				Actual	Predicted
		%		Nm	
1	20	2	3	70	91
2	30	3	5	330	343
3	20	2	3	85	91
4	20	1	3	190	149
5	30	1	5	325	332
6	10	3	5	340	331
7	30	2	3	220	182
8	30	3	1	300	298
9	20	2	3	75	91
10	20	2	1	165	142
11	10	3	1	340	346
12	20	2	3	70	91
13	10	2	3	210	200
14	10	1	5	305	320
15	20	3	3	175	168
16	20	2	5	190	165
17	10	1	1	320	319
18	20	2	3	75	91
19	30	1	1	250	271
20	20	2	3	72	91

(3) $$Size=90.8-9A+9.5B+11.5C+8AB+15AC-3.75BC+99.68A^{2}+67.182B^{2}+62.18C^{2}$$

**FIGURE 4:**
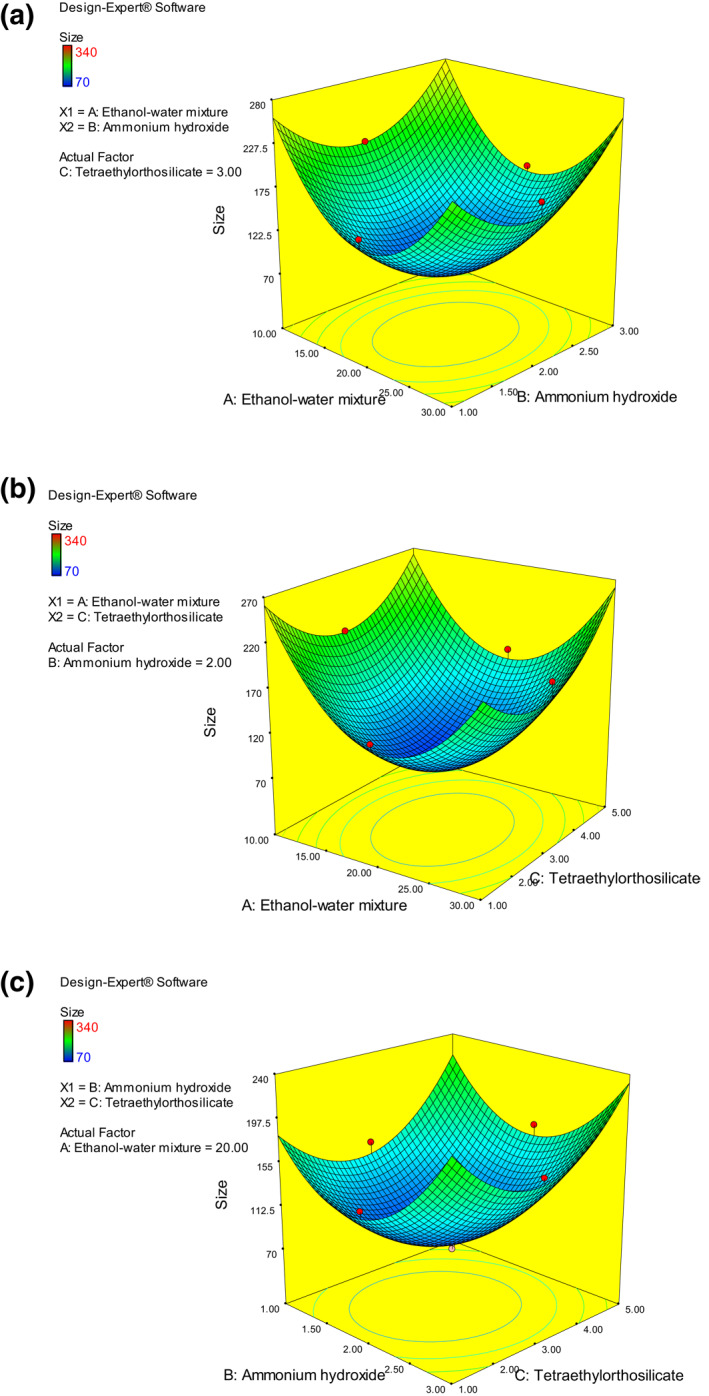
(a) B: Ammonium hydroxide versus A: Ethanol–water mixture. (b) A: Ethanol–water mixture versus C: Tetraethylorthosilicate. (c) B: Ammonium hydroxide versus C: Tetraethylorthosilicate, the response was evaluated as size of nanoparticles

Figure 4a–c shows that effective size of nanoparticle was obtained when ethanol‐water mixture, ammonium hydroxide and tetraethylorthosilicate at optimum concentration. The *R* ^2^ value was 96.45% compared with adjusted *R* ^2^ of 95.72% with respect to the predicted *R* ^2^ of 95.98% confirms the fitness of the test (Table 6).

**TABLE 6:** ANOVA for nanoparticle synthesis

Source	Sum of Squares	Df	Mean Square	F Value	*p*‐value Prob > F
Model	198,965.8	9	22,107.32	30.19833	<0.0001
A	810	1	810	1.10645	0.003176
B	902.5	1	902.5	1.232804	0.002928
C	1322.5	1	1322.5	1.806519	0.002086
AB	12.5	1	1852	1248	0.01425
AC	1800	1	1800	2.458778	0.001479
BC	112.5	1	112.5	0.153674	0.0033
A^2^	27,325.28	1	27,325.28	37.326	0.0001
B^2^	12,411.84	1	12,411.84	16.95442	0.0021
C^2^	10,633.09	1	10,633.09	14.52467	0.0034
Residual	7320.709	10	732.0709		
Lack of Fit	7163.209	5	1432.642	45.48069	0.2404
Pure Error	157.5	5	31.5		
Cor total	206,286.6	19			
Std. Dev.	27.05681		R‐Squared	0.964512	
Mean	205.35		Adj R‐Squared	0.957269	
C.V. %	13.17595		Pred R‐Squared	0.959823	
PRESS	43,108.53		Adeq precision	13.31773	

### 3.5 Preparation of functionalized silica nanoparticle‐doped chitinase (SiNp‐Chs)

**FIGURE 5:**
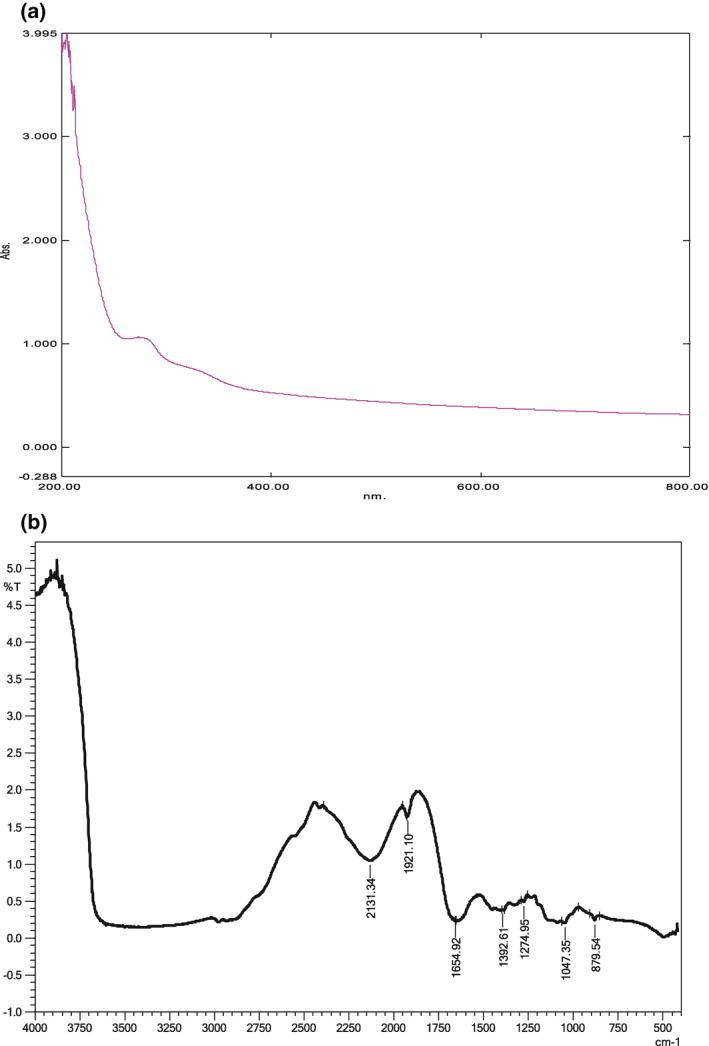
(a) UV Vis absorption spectra of synthesized SiNps –Chs. (b) FT‐IR spectra of SINp‐Chs

Sol–gel method was adopted to prepare functionalized SiNp‐Chs that primarily confirmed by UV visible spectroscopy and FT‐IR analysis. Figure 5a shows UV visible absorption spectra recorded from the synthesized SiNp‐Chs.

**FIGURE 6:**
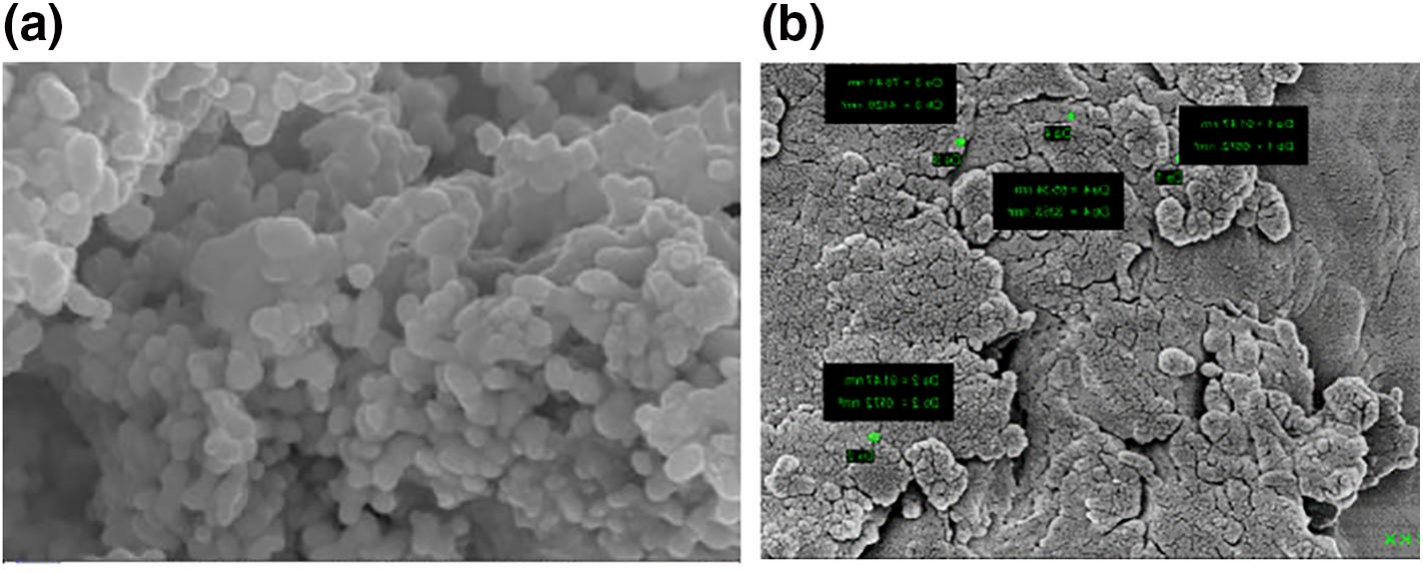
(a) SEM micrograph of SINp and functionalized SiNp‐Chs nano enzyme conjugate. (b) SiNp‐Chs nano enzyme conjugate with EDX spectra of SiNp‐Chs

Wavelength of the absorption band stabilizes at around 265 nm corresponds to protein (enzyme) and silica nanoparticles. Absorption band of the nano suspension is slightly asymmetrical with an indication of stabilizer. FT‐IR that is used to determine the functional groups, structure of a compound and purity of the sample in terms of frequencies of radiation. The profiles of FT‐IR spectroscopy of the SiNp‐Chs (Figure 5b) that reveals the main adsorption peaks at 1274.95, 1047.35, and 879.54 cm^−1^ are due to the asymmetric, symmetric and bending modes of SiO_2_, respectively. The absorption bands observed at 1921.1 and 2131.34 cm^−1^ are due to the bending of groups of functionalization agent 3‐amino propyl triethoxysilane (APS).The FT‐IR spectra show N‐H peaks at 2524 and 2653 cm^−1^, clearly indicating the organic modification of the nanoparticle surface. The absorbance of amide‐I at 1392.61 and1655 cm^−1^ to that of hydroxyl group at 3750 cm^−1^ is also observed. The differences in the vibration peaks is due to the specific interaction of functionalization agent APS –SiO_2_ with the chitinase functional groups. Further characterization of SiNp‐Chs using the SEM shows rough electron dense core shelled enlarged particles (80‐90 nm) in contrast to spherical, rough uniform dispersive‐free silica particles (80‐90 nm (Figure 6a,b).

**FIGURE 7:**
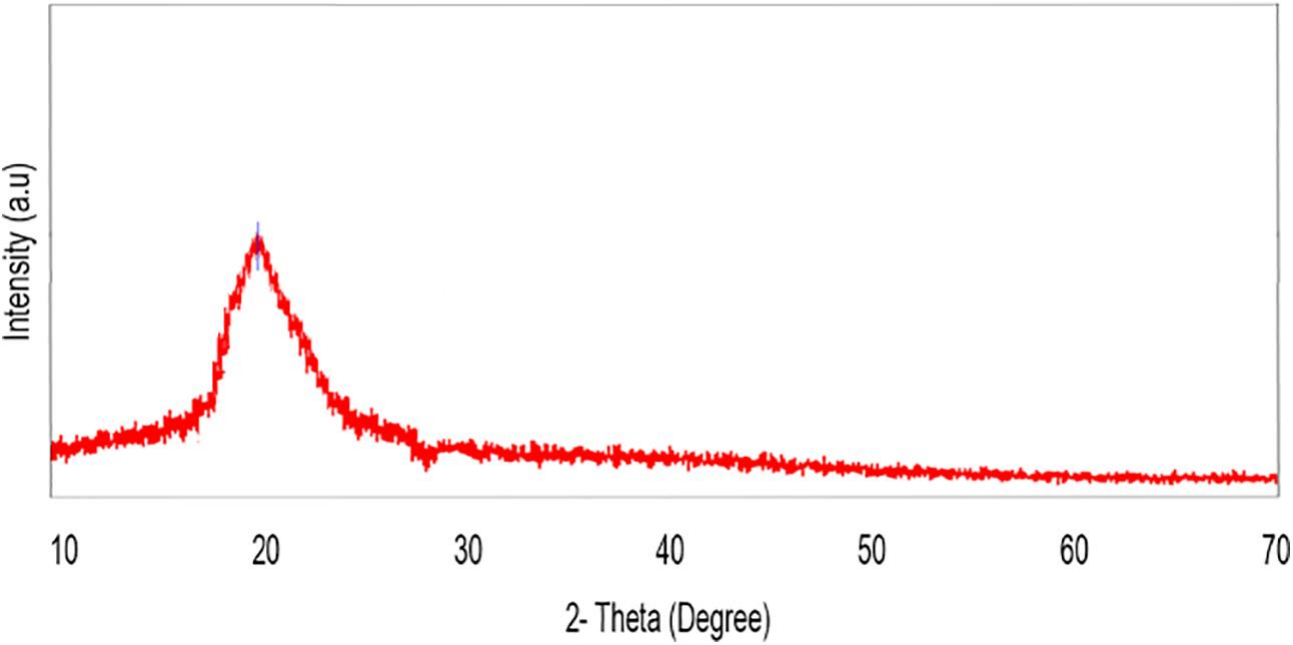
XRD pattern of SiNp‐Chs

The SEM analyser equipped with an EDX reveals a quantitative detection and localization of elements in the nano dispersive silica suggested by Stober and Fink [31]. The EDX images exhibited the presence of silica, carbon, nitrogen and oxygen. No characteristic peaks corresponding to silica crystalline structure was inferred from XRD analysis (Figure 7) that indicates amorphous nature of the formulation.

### 3.6 Pesticidal activity

Evaluation of pesticidal activity was done by food and contact toxicity methods against third and fourth instars of *S.litura* by determination of cumulative mortality, developmental influence, LT_50_ and LC_50_. SiNps‐Chs treatment caused higher impact than free SiNps on all the tested parameters.

#### 3.6.1 Free SiNp

**FIGURE 8:**
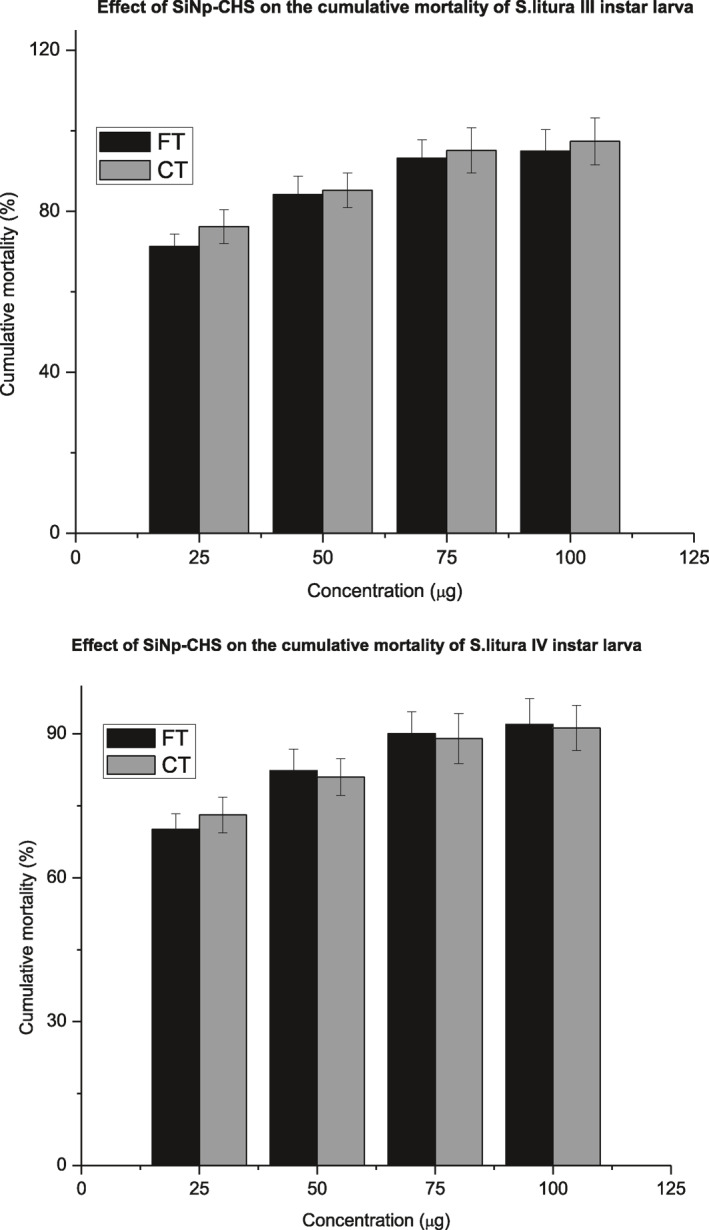
Effect of SiNp‐ChS on the cumulative mortality of *S.litura* III and IV instar larvae

Pesticidal activity of free SiNps was studied by contact and food toxicity assays with third and fourth instars of *S.litura*. Both instars that treated by both the assays were susceptible to the free SiNps as dose‐dependent manner. Though food toxicity method exhibited impact on the parameters, significant pesticidal activity was observed in contact toxicity (Figure 8).

High dosages of SiNps recorded maximum mortality against third and fourth instars. A gradual decline in the mortality rate was noticed in lower dosages of SiNp. As in cumulative mortality, life stage parameters were influenced by high dosages of free SiNp. Maximum reduction of larval, pupal period, adult emergence and adult longevity brought about by high dosages of free SiNps. Dosage‐dependent variation on the LT_50_ and LC_50_ was also recorded. High dosages of SiNps showed lower LT_50_ against the both instars. The results of LC_50_ values were shown in the Table 7 that determined through Probit analysis. It is very clear that the LC_50_ values of 3rd and 4th larval instars of *S.litura* in response to different dosages showed an increased trend in the LC_50_ value when the dosage of the nanoformulation was decreased.

**TABLE 7:** Effect of F‐SiNp and SiNP‐Chs on LC_50_ parameters of *S.litura*

Treatment	Instars	Regression equation *Y* = *a* + *bx*	LC50 (µg)	Variance	Chi‐square Value	Fiducial limit (95% confidence)	
						Upper	Lower
Free SiNp	I	‐1.24 + 1.1	1.31	0.21	1.11	1.41	0.12
	II	‐2.11 + 3.11	4.32	0.63	2.01	5.03	0.31
	III	0.1 + 1.23	12.12	0.71	3.11	6.13	3.01
	IV	0.21 + 3.02	20.31	0.83	5.13	8.01	5.21
	V	0.9 + 6.11	47.12	1.03	7.31	9.03	6.01
	VI	1.21 + 3.11	81.41	3.12	9.21	12.0	7.01
Free SiNp	I	‐1.12 + 0.2	0.24	0.34	2.31	1.31	0.23
	II	‐1.14 + 2.12	2.11	0.41	3.01	3.03	1.31
	III	0.2 + 1.01	8.01	0.63	5.12	5.21	3.03
	IV	0.4 + 2.43	12.23	0.91	7.32	7.21	4.14
	V	0.7 + 5.13	27.01	2.13	8.21	8.01	5.04
	VI	0.9 + 4.12	62.12	4.21	9.33	9.10	7.04

The mechanism of control of insect pest using nano silica is based on the fact that insect pests used a variety of cuticular lipids for protecting their water barrier and thereby prevent death from desiccation. Nanosilica after contact with the insects rapidly adsorbed into the cuticular lipid followed by complete degeneration or rupture of cuticle, leads to death [24].

#### 3.6.2 SiNp‐Chs

**FIGURE 9:**
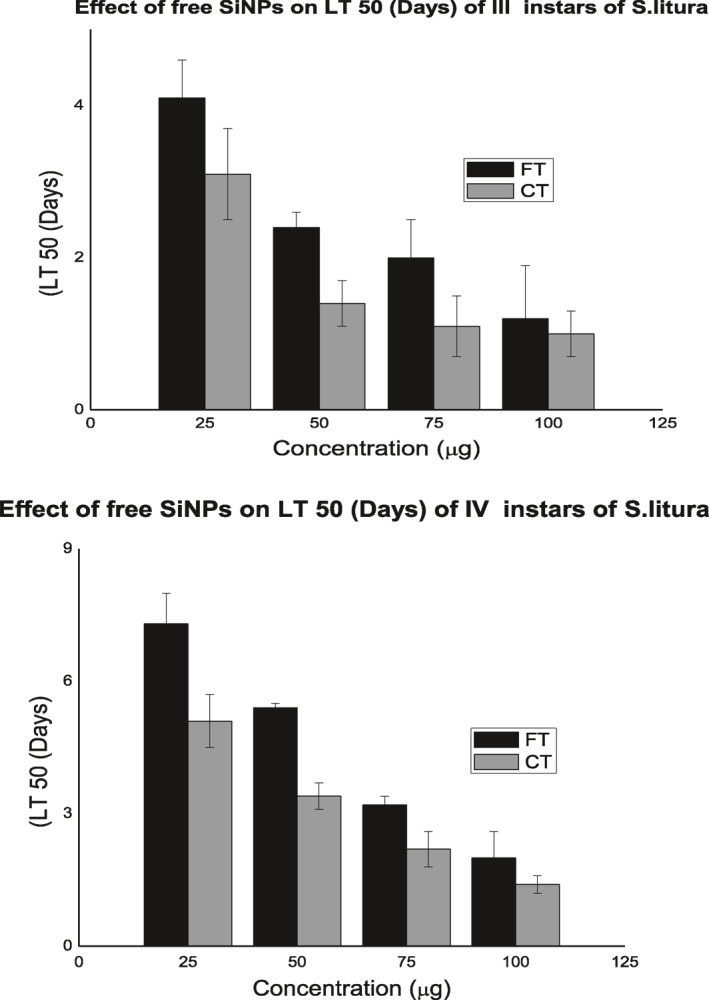
Effect of SiNp‐ChS on the LT50 of *S.litura* III and IV instars larvae

Significant effect on pesticidal activity was recorded in SiNp‐Chs treatment against both the tested instars that treated with the both assays. All the tested concentration of SiNp‐Chs exhibited high rate mortality, drastic reduction of life stage parameters as non‐dose‐dependent manner. SiNp‐Chs recorded lower LC_50_ and LT_50_ than free SiNp against both the tested instars (Figure 9). Enhanced activity of SiNp‐Chs is due to sustained or controlled release pattern of Chs from the functionalised SiNp nano formulation. Chitinase—a hydrolytic enzyme that catalyses hydrolysis of chitin to its monomer *N*‐acetyl‐d‐glucosamine chitin forms the exoskeleton of most of the invertebrates [15,16]. Hydrolytic action of chitinase weakened the exoskeleton that might facilitate rapid adsorption of nanosilica into cuticular lipid followed by complete degeneration or rupture of cuticle, leads to death.

### 3.7 Preparation of biopesticidal plant extract‐loaded SiNp‐Chs

Plant‐based biopesticides (botanicals) are being extensively used as a component of IPM [25]. It has already been reported that plant species possessing pest control properties included 1005 species with anti‐feedent, 1297 species with repellent, 27 species with attractant and 31 species with growth inhibition properties [32]. Recent studies shows that biopesticides based on plant extracts are promising candidates for the effective control insect pests. Despite the pesticidal effects, plant‐based biopesticides lose their effects against environmental condition. Recent studies shows that nanoformulation of plant‐based biopesticidal agents with biocompatible metallic and non‐metallic nanoparticles preparation protect or improve the pesticidal efficiency of pesticidal phytochemicals without affecting non‐target organisms. With this objective, herein, it is undertaken to evaluate the enhanced or improved pesticidal activity of biopesticidal plant extracts loaded with silica nanoparticles loaded with chitinase (SiNp‐Chs) against *S.litura* was studied. Loading of ethanolic extract of biopesticidal plants with SiNp‐Chs was done to determine enhanced pesticidal activity. Confirmation of effective doping of active phytochemicals of respective plant extracts with functionalized SiNp‐Chs was done by UV visible spectroscopy analysis.

Loading of the bioactive phytochemicals with SiNp‐Chs was primarily confirmed by monitoring changes in the absorption peaks with UV–Vis spectroscopy.

**FIGURE 10:**
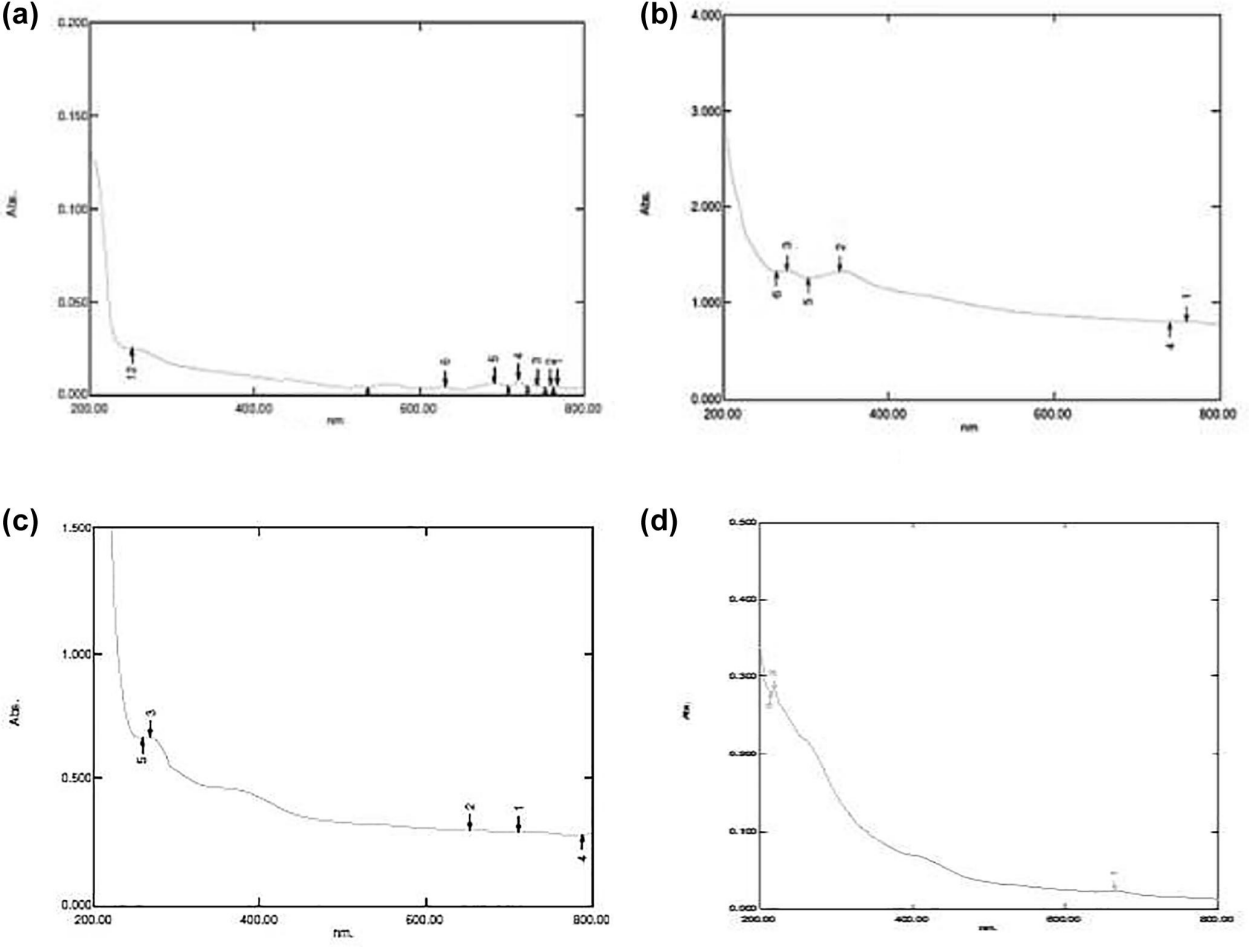
UV Visible absorption spectra of SiNp‐Chs‐loaded (a) *Adhatoda vasica*, (b) neem, (c) *Leucas aspera* and (d) turmeric

Figure 10 reveals the UV‐vis spectra recorded from the plant extract‐loaded nano formulation. After loading of all the plant extracts, the wavelength of the surface plasmon band stabilizes at 275 that correspond to SiNps absorption band is slightly asymmetrical with indications of loading of phytochemicals components of the respective plant extracts at 380–610 nm.

### 3.8 Evaluation of enhanced pesticidal activity

**FIGURE 11:**
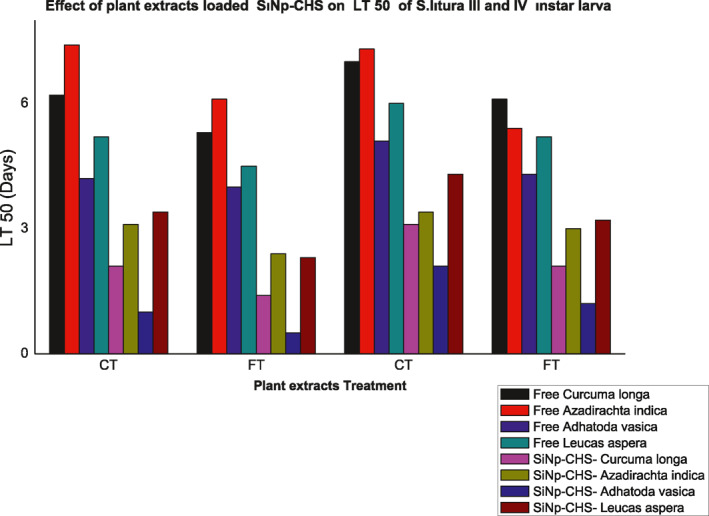
Release profile of plant extract‐loaded SiNp‐Chs

Enhanced pesticidal activity of SiNps‐Chs loaded with respective plant extracts against third and fourth instars of *S.litura* also studied by food and contact toxicity tests as described earlier. Both the toxicity tests supported enhanced pesticidal activity by showing high mortality, reduced developmental period, LC_50_ and LT_50_ parameters. Among the plant extract‐loaded SiNp‐Chs, neem extract—SiNp‐Chs formulation showed maximum impact on all the tested parameters on the both tested instars. Complete mortality of 3rd and 4th instars was observed in all the tested dosages of neem extract—SiNp‐Chs except 10 µg and the same formulation brought about complete absence of pupal and adult emergence. Enhanced pesticidal activity of fungal metabolites with chitosan nanoparticles against *S.litura* reported by Bharani et al. who observed the cumulative mortality of *S.litura* was found to be increased in nano formulation treatment (Figure 11).

Enhanced acaricidal essential oil derived from *Achillea millefolium* loaded with chitosan nanocapsules against adult *Tetranychus urticae* Koch is recently reported [33].

Nano‐encapsulation of active agrochemicals allows proper absorption of the phytochemicals into the plants due to slow and sustained release and has a long lasting and persistent effect unlike the bulk agrochemicals [3,34]. Improved pesticidal activity of SiNp‐Chs‐plant extracts treatment in this study might be due the sustained or controlled release of pesticidal phytochemicals from nano‐encapsulated plant extracts.

Effect of SiNp‐Chs loading on the release of pesticidal metabolites was studied by determination of cumulative mortality of 3rd instar of *S.litura* adopting food and contact toxicity tests as described earlier. Sustained or controlled release of pesticidal metabolites from the nano enzyme conjugate was confirmed by a gradual increase in the rate of cumulative mortality at the respective time periods that would provide continuous effect against larval instars. In the both assays, maximum mortality of the larval instars was observed at 96 h.

### 3.9 Brine shrimp toxicity assays

Biocompatibility assessment of nanoformulation was studied by Brine Shrimp Toxicity Lethality Assay that is a convenient system for monitoring biological activities. It is a very useful method for the assessment of the toxic potential of various compounds [33]. *Artemia* is one of the most valuable test organisms available for ecotoxicity testing, and the available research suggests that several applications of *Artemia* to toxicology and ecotoxicology will continue to be used widely [11]. With this objective, herein, it is undertaken to evaluate nanoformulation‐mediated toxic effect on *A.salina* by determination of cumulative mortality and protein profiling studies. Figure depicts the cumulative mortality of *A.salina* exposed to different concentrations of nanoformulation that revealed no significant mortality against *A.salina* at all the tested concentration of free silica nanoparticles except 100 µg dosage that exhibited 41.2%. In contrast, nano enzyme conjugate at high concentration recorded 32.0 % mortality. No mortality was observed in all the tested concentration of free chitinase treatment. Toxicity of respective treatment against *A.salina* was further confirmed by lethal dose 50 (LD_50_) determination using Probit analysis. The results clearly show LD_50_ of free silica nanoparticles and nano enzyme conjugate were found to be 155 and 95 µg, respectively.

### 3.10 Protein profile studies

**FIGURE 12:**
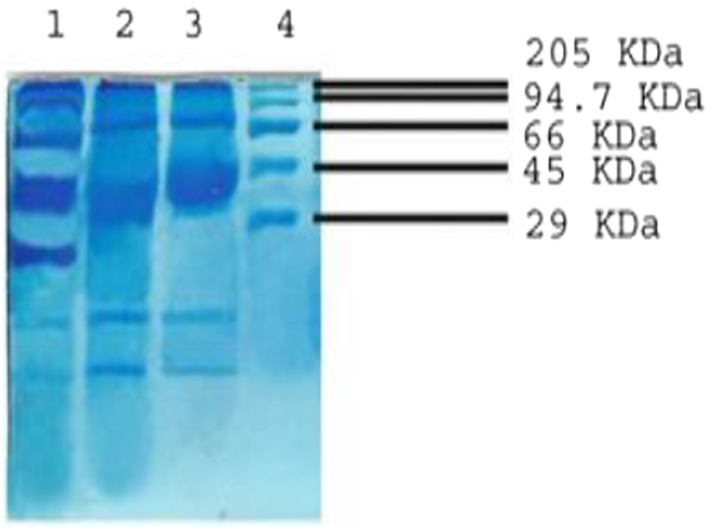
SDS PAGE. Lane‐1: *Artemia* in response to nano enzyme conjugate exposure. Lane‐2: *Artemia* in response to nanosilica exposure. Lane‐3: *Artemia* in response to nanosilica and nano enzyme conjugate exposure. Lane‐4: Molecular Marker

Although the adverse effects of nanomaterials on marine organisms have been widely studied, their effects at the molecular level are poorly understood. Accumulated ROS subsequently elicits several biological responses such as oxidative stress‐induced signalling pathways, apoptosis and inflammation. We focused mainly on protein profile of the *Artemia* in response to nanosilica and nano enzyme conjugate exposure. The SDS‐PAGE analysis of proteins in treated *Artemia* showed a stress‐induced protein with a molecular weight of 80 kDa (Figure 12).

This protein band was not seen prominently in the control sample. The toxicity of nano enzyme conjugate in cells mainly arises from oxidative stress via the generation of ROS, resulting in cellular physiology effects. Taken together, the induction of new protein was involved in nano enzyme conjugate‐induced oxidative stress, implying that these processes are likely to be an important defence mechanism against nanomaterial‐induced oxidative stress in *Artemia salina*.

## 4 CONCLUSION

Principles of nanoscience and nanotechnology is currently utilized in agriculture sector rather than medicine and health care sector for the improved delivery of nutrients, insect pests and pathogens control and rapid diagnosis of diseases. Pesticidal activity of silica nanoparticle‐loaded chitinase nano drug conjugate and its synergistic effect with pesticidal plant extracts against *S.litura* was done. Nano enzyme conjugate prepared in this study recorded enhanced pesticidal activity and best compatibility with pesticidal plant extracts. Toxicity lethal study against brine shrimp (*Artemia salina*) exhibited the distinct biosafety that all implies that the prepared nano enzyme conjugate can be used as an effective, safe pesticidal agent against economic important insect pests.

## References

[1] Barik, T.K. , Sahu, B. , Swain, V. : Nanosilica—from medicine to pest control. Parasitol. Res. 103(2), 253–256 (2008) 18438740 10.1007/s00436-008-0975-7

[2] Bhattacharyya, A. , et al.: Nanoparticles ‐A recent approach to insect pest control. Afr. J. Biotechnol. 9(24), 3489–3493 (2010)

[3] Christofoli, M. , et al.: Insecticidal effect of nanoencapsulated essential oils from Zanthoxylum rhoifolium (Rutaceae) in Bemisia tabaci populations. Ind. Crop. Prod. 70, 301–308 (2015)

[4] Derosa, M.C. , et al.: Nanotechnology in fertilizers. Nat. Nanotechnol. 5, 91–94 (2010) 20130583 10.1038/nnano.2010.2

[5] Ehdaie, B. : Application of nanotechnology in cancer research: Review of progress in the National Cancer Institute's Alliance for Nanotechnology. Int. J. Biol. Sci. 3, 108–110 (2007) 17304339 10.7150/ijbs.3.108 PMC1796952

[6] Finney, D.J. : The adjustment for a natural response rate in probit analysis. Ann. Appl. Biol. 36, 187–195 (1949) 18151945 10.1111/j.1744-7348.1949.tb06408.x

[7] Goswami, A. , et al.: Novel applications of solid and liquid formulations of nanoparticles against insect pests and pathogens. Thin Solid Films. 519, 1252–1257 (2010)

[8] Hallberg, K. : Towards a responsible research in nanoscience and nanotechnology PASI NANO‐BIO, pp. 1–47 (2010)

[9] Harper, S. : New approaches needed to gauge safety of nanotech‐based pesticides. Res. Urge’ Phys. Chem. 4(33), 2010–2012 (2010)

[10] Hussaini, A. , Mahasneh, M. : Microbial growth and quorum sensing antagonist activities of herbal plants extracts. Molecules. 14, 3425–3435 (2007) 10.3390/molecules14093425 PMC6255472 19783935

[11] Insanu, M. , Anggadiredja, J. , Kayser, O. : Curcacycline A and B–new pharmacological insights to an old drug. Int. J. Appl. Res. Nat. Prod. 5, 26–34 (2012)

[12] McLaughlin, J.L. , et al.: American Chemical Society, Washington, DC, pp. 112–137 (1993)

[13] Chakravarthy, K. , et al.: DNA‐tagged nano gold: a new tool for the control of the armyworm, Spodoptera litura Fab. (Lepidoptera: Noctuidae). Afr. J. Biotechnol. 11(38), 9295–9301 (2012)

[14] Khot, L.R. , et al.: Applications of nanomaterials in agricultural production and crop protection: A review. Crop Protec. 35, 64–70 (2012)

[15] Liu, Y. , Tong, Z. , Prud'homme, R.K. : Stabilized polymeric nanoparticles for controlled and efficient release of bifenthrin. Pest Manag. Sci. 64, 808–812 (2008) 18366056 10.1002/ps.1566

[16] Liu, B.L. , et al.: Production of chitinase from Verticillium lecanii F091 using submerged fermentation. Enzyme Microb. Technol. 33, 410–415 (2003)

[17] Perez‐de‐Luque, A. , Rubiales, D. : Nanotechnology for parasitic plant control. Pest Manag. Sci. 65, 540–545 (2009) 19255973 10.1002/ps.1732

[18] Peteu, S.F. , et al.: Responsive polymers for crop protection. Polymers. 2, 229–251 (2010)

[19] Yang, F.L. , et al.: Structural characterization of nanoparticles loaded with garlic essential oil and their insecticidal activity against Tribolium castaneum (Herbst) (Coleoptera: Tenebrionidae). J. Agric. Food Chem. 57, 10156–10162 (2009) 19835357 10.1021/jf9023118

[20] Arvind Bharani, R. , Karthick Raja Namasivayam, S. , Sai Shankar, S. : Biocompatible chitosan nanoparticles incorporated pesticidal protein Beauvericin (Csnp‐Bv) preparation for the improved pesticidal activity against major Groundnut defoliator Spodoptera litura (Fab.) (Lepidoptera; Noctuidae). Int. J. Chem. Tech. Res. 6(12), 5007–5012 (2014)

[21] Bharani, R.A. , Namasivayam, S.K.R. : Biogenic silver nanoparticles mediated stress on developmental period and gut physiology of major lepidopteran pest Spodoptera litura (Fab.) (Lepidoptera: Noctuidae)—an eco‐friendly approach of insect pest control. J. Environ. Chem. Eng. 5(1), 453–467 (2017)

[22] Narendrakumar, G. , et al.: Enhancement of biocontrol potential of biocompatible bovine serum albumin (BSA) based protein nanoparticles loaded bacterial chitinase against major plant pathogenic fungi Alternaria alternata. Biocatalysis Agric. Biotechnol. 15, 219–228 (2018)

[23] Rajabi, S. , et al.: Artemia salina as a model organism in toxicity assessment of nanoparticles. J. Pharmaceut. Sci. 23, 20 (2015) 10.1186/s40199-015-0105-x PMC4344789 25888940

[24] Teodoro, S. , Micaela, B. , David, K.W. : Novel use of nano‐structured alumina as an insecticide. Pest Manag. Sci. 66(6), 577–579 (2010) 20127753 10.1002/ps.1915

[25] Rogerio, A. , et al.: Compatibility of the Fungus Beauveria bassiana (Bals.) Vuill. (Deuteromycetes) with extracts of neem Seeds and leaves and the emulsible. Oil. Neotrop. Entomol. 34(4), 601–606 (2005)

[26] Ahmadi, Z. , et al.: Achillea millefolium essential oil and chitosan nanocapsules with enhanced activity against Tetranychus urticae. J. Pest Sci. 91, 837–848 (2018)

[27] Roy, D. , Cambre, J.N. , Sumerlin, B.S. : Future perspectives and recent advances in stimuliresponsive materials. J. Prog. Polym. Sci. 35, 278–30 (2010)

[28] Sabbour, M.M. : Entomotoxicity assay of Nano‐particle 3‐(Zinc oxide ZnO) against Sitophilus oryzae under laboratory and store conditions in Egypt. J. Sci. Res. Rep. 1(2), 50–58 (2013)

[29] Shrivastava, S. , et al.: Characterization of enhanced antibacterial effects of novel silica nanoparticles. Nanotechnology. 18, 225103 (2007) 10.1088/0957-4484/18/22/225103 37016550

[30] Stadler, T. , Butelerb, M. , Weaver, D.K. : Novel use of nanostructured alumina as an insecticide. Pest Manag. Sci. 66, 577–579 (2010) 20127753 10.1002/ps.1915

[31] Stober, W. , Fink, A. : Controlled growth of monodisperse silica spheres in the micron size range. J. Colloid Interface Sci. 26, 62–69 (1968)

[32] Sahayaraj, K. , Karthick Raja Namasivayam, S. , Martin Rathi, R. : Compatibility of entomopathogenic fungi with extracts of plants and commercial botanicals. Afr. J. Biotechnol. 10(6), 933–938 (2011)

[33] Shen, C.X. , et al.: Induction of programmed cell death in Arabidopsis and rice by single‐wall carbon nanotubes. Am. J. Bot. 97, 1602–1609 (2010) 21616795 10.3732/ajb.1000073

[34] Torres, E. , et al.: Gold nanoparticle formation by seaweed biomass: influence of pH. NanoBiotechnology. 14–17 (2005)

